# The Persistent Power of the Taxane/Platin Chemotherapy

**DOI:** 10.3390/cancers17071208

**Published:** 2025-04-02

**Authors:** Lucy B. Xu, Elizabeth R. Smith, Vasili Koutouratsas, Zhe-Sheng Chen, Xiang-Xi Xu

**Affiliations:** 1 Department of Biology, University of Miami, Miami, FL 33136, USA; lxx152@miami.edu; 2 Sylvester Comprehensive Cancer Center, Miller School of Medicine, University of Miami, Miami, FL 33136, USA; 3 Department of Obstetrics, Gynecology and Reproductive Sciences, Miller School of Medicine, University of Miami, Miami, FL 33136, USA; 4 College of Pharmacy and Health Sciences, St. John’s University, Queens, NY 11439, USA; vasili.koutouratsas20@my.stjohns.edu (V.K.);; 5 Department of Radiation Oncology, Miller School of Medicine, University of Miami, Miami, FL 33136, USA

**Keywords:** chemotherapy, taxanes, taxol, paclitaxel, microtubules, mitosis, proliferation, cisplatin, carboplatin, nuclear envelope, micronuclei, DNA damage, programed cell death, apoptosis, nuclear membrane rupture, drug resistance, drug mechanisms

## Abstract

The cancer chemotherapy regimen of a taxane and platinum combination was developed more than forty years ago, yet remains the cornerstone of treatment for several major cancer types today. Although many new agents targeting cancer genes and pathways have been developed and evaluated, none have been sufficient to replace the long-established taxane/platinum combination. This leads us to ponder why, after four decades of colossal efforts, multiple discoveries, and tremendous advances in understanding gene mutations and biology, the development of conceptually superior targeted therapies has not yet achieved overwhelming success in replacing cytotoxic chemotherapy. The concept of targeted therapy is based on the idea that blocking the altered pathway(s) crucial for cancer development (and maintenance), the disturbance in cellular signaling, metabolism, and functions will make the targeted cancer cells unfit and trigger programmed cell death in cancer cells, but without the significant side effects that limit chemotherapy. We propose that the lack of anticipated triumphs of targeted therapy stems from the desensitization of programmed cell death pathways during neoplastic transformation and malignant progression of cancer cells. This renders the targeting drugs largely ineffective at killing cancer cells and mostly insufficient in clinical implements. Recent advances in understanding suggest that, in contrast to targeted therapies, taxanes and platinum agents kill cancer cells by physical rupturing nuclear membranes rather than triggering apoptosis, making their effect independent of the intrinsic cellular programmed cell death mechanism. This new recognition of the non-programmed cell death mechanism in the success of chemotherapeutic agents, such as taxanes and platinum, may inspire oncologists and cancer researchers to focus their efforts more productively on developing effective non-programmed cell death cancer therapies to replace or significantly improve the application of the current standard taxane/platinum regimens.

## Simple Summary

Cancer chemotherapy regimen of taxanes and platinum combination was developed more than forty years ago, and yet remains the cornerstone of treatment for several major cancer types today. We suggest that the persistent power of taxanes and platinum agents is accounted for by the newly found cellular action of these drugs by physical rupture of nuclear membranes rather than triggering apoptosis, independent of the intrinsic cellular programmed cell death mechanism. This new recognition of non-programmed cell death mode of action in the successes of chemotherapeutic agents, taxanes and platinum, may inspire a more fruitful direction to develop effective non-programmed cell death cancer therapies.

## 1. Introduction

Cancer chemotherapy, particularly using the taxane (paclitaxel/docetaxel/carbozitaxel) and platinum (cisplatin/carboplatin/oxaliplatin) combination, was developed over forty years ago and is still the cornerstone of cancer treatment today [1,2,3,4,5]. However, the development of drug resistance, along with substantial and accumulative side effects, limits the application of the regimen. Targeted therapy that, in theory, has minimal toxic side effects on non-cancer cells, would be a superior treatment. The concept of targeted therapy is that by blocking the altered pathway(s) crucial for cancer development and maintenance, the disturbance in cellular metabolism and functions will trigger programmed cell death of the cancer cells. Although many new agents targeting cancer genes and pathways have been developed and evaluated, none is sufficient to replace the long-established taxane/platinum combination. This leads us to ponder why our efforts to develop conceptually superior targeted therapies, based on many biological discoveries and new understandings of cancer mutations over the years, has not yet achieved overwhelming success in replacing cytotoxic chemotherapy.

We propose that the desensitization of programmed cell death pathways and processes, acquired during the malignant progression of cancer cells, makes the targeted drugs ineffective.

In contrast, recent advances in understanding show that taxanes and platin agents kill cancer cells by physical rupturing nuclear membranes, rather than the long-thought mechanism of apoptosis, independent of the intrinsic cellular programmed cell death mechanism.

The new recognition of the non-programmed cell death mechanism in the success of chemotherapeutic agents may inspire oncologists and cancer researchers to focus their effects in a more fruitful direction, developing effective future cancer therapies to replace the current standard taxane/platinum regimen. Here, we discuss these issues to formulate our arguments.

## 2. Historic Overview and Current State of Cancer Treatment

Historically, the development of a successful cytotoxic chemotherapy began with the treatment of leukemia using mustard gas (sulfur or nitrogen mustard), inspired by the pathological observation of victims exposing to this toxic agent, which was designed as a chemical weapon during World War I [6,7,8,9,10]. Mustard gas poisoning caused severe damage to blood and bone marrow in victims. The distinctive toxicity to blood cells prompted the testing of nitrogen mustards, first in animal lymphoid tumors and then in patients, to counter leukemia. In the early efforts (1940–1950), Louis Goodman, Alfred Gilman, and colleagues at the Yale School of Medicine reasoned, attempted, and reported the treatment of a patient with lymphosarcoma using tris-nitrogen mustard [6,7,8,9,10]. Tumor regression was observed, though it lasted only a few weeks. However, this limited ability to counter cancer was enough to dispel the looming pessimism of the time and encourage additional attempts.

The concept of using toxic compounds to treat cancer became an early form of intervention, known as chemotherapy, and various toxic agents and their combinations were tried and progressively improved [11,12,13,14,15]. The possibility of using chemicals to suppress cancer was encouraging, leading to increased investment in exploring this method following the establishment of the Cancer Chemotherapy National Service Center in 1955. This, in turn, sparked enthusiasm and the development of new cytotoxic compounds to treat cancer [11,12,13,14,15]. This enthusiasm peaked in the 1960s and early 1970s, when reports of cures for acute childhood leukemia and Hodgkin’s lymphomas emerged, following combination chemotherapy using an increasing number of newly discovered drugs from screening programs [14,16,17].

Notable new drugs subsequently developed include an antifolate compound reported by Sidney Farber [18], as well as additional anti-metabolite compounds of purine and pyrimidine analogs, and additional cytotoxic molecules including imidizole compounds, vincarosea alkaloids, camptothecin analogs, platinum compounds, and taxols/taxanes [10,11,19,20]. Some of these compounds became key agents in cancer management, particularly using taxane (paclitaxel/docetaxel/carbozitaxel) and platin (cisplatin/carboplatin/oxaliplatin) combinations. The chemotherapy regimen developed over forty years ago is still the unsurpassed optimal and frontline cancer treatment today.

A number of articles have recounted the conception and trials of earlier concepts, as well as the progress that made chemotherapy a cornerstone of cancer treatment today [6,7,8,11,13,14,15,19,20]. The discoveries and application of cisplatin (and later carboplatin) and paclitaxel/taxol (the first taxanes) both occurred by chance as serendipitous observations and findings, rather than rational research and drug design [21,22].

The discovery of platinum compounds as a major chemotherapeutic agent was surprising and fortuitous [1,3,23]. The accidental discovery of cisplatin as an anti-cancer drug is a remarkable story, often described from a personal perspective and experience [24,25,26]. In the 1960s, a physicist, Barnett Rosenberg in the Biophysics Department of Michigan State University, set out to determine if electric fields would interfere with mitosis [3,24,25,26]. The first experiment, using the electric field device designed to test the question, produced interesting results, showing that the treated E. coli became filament in morphology. This suggested that growth was not suppressed but division was blocked. Treatment with the electric field device also stopped mitosis of mammalian tumor cells [3,23,27].

Working with biologist Loretta Van Camp, it took several years to determine that it was a chemical agent, rather than the electric field, that gave the mitotic inhibitory activity. The active agent was found to be cisplatin, produced by the device [3,28,29]. The persistent effort and personal endeavor of Dr. Barnett Rosenberg and his colleagues made this unlikely agent a common cancer drug. The success of cisplatin in the treatment of testicular cancer by Dr. Lawrence Einhorn at the University of Indiana Medical School sparked enthusiasm and led to further development and testing of the agent for cancer treatment. Furthermore, cisplatin was found to be active against other cancer types such as ovarian, lung, etc. [3], establishing the standardized chemotherapeutic protocols still used today. Carboplatin was developed later and is preferred because it is less toxic than cisplatin. Today, a combination of paclitaxel and carboplatin is generally used as the frontline treatment of ovarian cancer [4,30].

Extensive studies of platinum drugs have been conducted [1,3,31,32,33]. Currently, the mechanistic action for platinum cytotoxicity is generally thought to be the formation of DNA adducts between platinum and cellular components such as DNA and various proteins [3,31,32,33,34]. However, the mechanism for their efficacy is still murky.

Paclitaxel/Taxol, the first taxane drug, originated from a screening project of natural compounds extracted from plants, initiated by the USDA [35]. The unlikely path to the discovery and development of Taxol/paclitaxel has been described in many articles [5,35,36,37]. Several key events, such as the identification of tumor inhibitory activity from barks of the Pacific yew tree, identification of molecular structure, determination of activity in microtubule stabilization, and successful clinical trials, propelled taxanes to become a key group of drugs used to treat a wide range of major solid tumors today [5,21,22,35,36,37].

In clinical application, paclitaxel was first found to be active for ovarian cancer [38,39,40,41] and was subsequently tested and found to be active in several other major solid tumor types [21,22]. Currently, a number of taxanes (such as docetaxel and carbozitaxel) join paclitaxel, the first taxane, in acting through a mechanism of microtubule stabilization. These drugs are commonly used to treat ovarian, breast, lung, and prostate cancer [22,42].

In the 1990s, the combination of cisplatin and paclitaxel as a chemotherapeutic regimen to treat ovarian cancer was established [30,43,44,45,46]. Since then, the taxane/platin combination has become the cornerstone of frontline treatment for most of the major solid tumor types [21,22]. The regimen is highly successful and persists in being the option for frontline treatment for many solid tumors today, and will likely be used for the foreseeable future.

Nevertheless, side effects of chemotherapy are a constant issue associated with cisplatin and paclitaxel regimen, and an obvious alternative concept is that targeting a cancer-specific mutation/alteration would have few side effects and might be a superior strategy [34,47].

Both the commonly used taxanes and platinum agents were products of serendipitous discovery, though their successes have surpassed expectations. Nevertheless, their cancer-killing mechanisms are still being investigated and worked out [1,3,48,49]. The rapid progress in understanding cell and cancer biology by the scientific community has provided the knowledge and feasibility to design and develop rationally based drugs and therapies that may be highly effective and non-toxic. Indeed, in recent years, great effort has been devoted to understanding and developing targeted therapies—which are, as implied by the name, drugs that modulate targets specifically associated with cancer but not normal cells. The history for the progressive development of cancer therapy is comprehensively described in “The Emperor of All Maladies: A Biography of Cancer”, a book by Siddhartha Mukherjee in 2011 [50].

## 3. Challenges of Targeted Therapy

**Figure 1:**
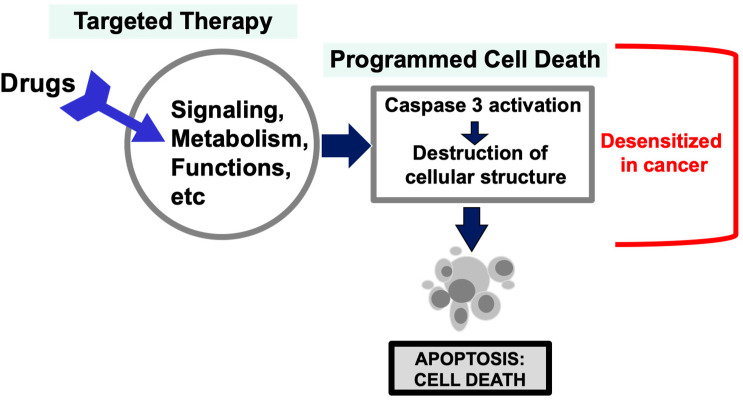
Cell death mechanisms. Concept of the activation of programmed cell death pathway in cancer therapies. An illustration of the concept that anti-cancer drug/agents activate steps leading to activation of a step in the cellular regulatory pathway leading to programmed cell death is presented. By targeting tumor suppressor or oncogenes and pathways altered in neoplastic cells, cancer drugs may interfere with cellular signaling, metabolisms, functions such as mitosis, etc., which will reduce cell fitness and survival. Anti-cancer drugs/agents are generally thought to kill cancer cells by activating an intrinsic programmed cell death pathway, involving the leaking of mitochondrial cytochrome C, assembly of Apaf-1 into apoptosomes, activation of caspase 9, and subsequently caspase 3, leading to widespread proteolytic destruction of proteins and cellular structure, and ultimately cell death. However, an idea is that the apoptotic programmed cell death pathway is desensitized during transformation and development of cancer, and cancer cells have a higher threshold to initiate apoptosis than normal cells. This will be an immense obstacle for the success and efficacy of targeted therapeutic drugs.

This leads us to ponder why our efforts to develop conceptually superior targeted therapies based on many discoveries and understanding of cancer mutations over the years have not yet achieved overwhelming success in replacing chemotherapy. The reasoning behind targeted therapy is based on the idea that by blocking the altered pathway(s) crucial for cancer development, the disturbance in cellular metabolism and functions will trigger the intrinsic programmed cell death of the cancer cells [51,52,53,54,55,56,57,58,59] (Figure 1).

With the discoveries and understanding of the critical roles of the many tumor suppressor genes and oncogenes in cancer development [55,60], their roles in cancer maintenance have also been demonstrated in laboratory settings and mouse models. A good number of seemingly excellent genes have been identified as apparently suitable targets for cancer therapy. Many examples of targeting these genes have been attempted and are still being actively pursued. Outstanding examples are as follow: the restoration of Tp53 conformation and function by small molecular drugs [61]; the blockage of Ras signaling by the farnesyl transferase inhibitors (FTI) [62,63]; the ongoing development of Ras inhibitors, the various inhibitors for EGFR, MEKs, and Erks, components of the Ras signaling pathway [64]; additional oncogenic kinases such as AKT, PI3K, etc., the survival pathways, and Bcl-2 inhibitors to sensitize the apoptotic pathway; cyclin-dependent kinase inhibitors (CDKs) to block cancer cell cycle. These are just a few examples. Successful cases are well known, such as Herceptin for inhibiting Her2/NEU [65,66,67], CDK4/6 inhibitors used in breast cancer treatment [68,69,70], and imatinib (Gleevec), a celebrated success of the targeting strategy for treating chronic myeloid leukemia [71,72]. However, overwhelming success has not been achieved, as most solid tumors are still treated with the standard chemotherapy regimens.

A particular example of targeted therapy is the efforts in targeting Ras, which mutates in nearly half of cancer, and would be an apparently great Achille’s heel for cancer cells [73,74,75,76]. Through the technological breakthrough from the efforts of the RAS Initiative, and the focused work at Frederick National Laboratory for Cancer Research, the “undruggable” Ras genes were addressed, and effective and specific molecules, some targeting the only the mutated Ras oncoprotein, were developed [74,75,76]. These magic drugs were the achievement of keen scientific minds and collective endeavors by the scientific communities. Anti-cancer activity was demonstrated, though the drugs are not “silver bullets” [77,78,79], and the latest studies recommend that the Ras drugs may enhance chemotherapy, but on their own, they lack sufficient efficacy to impact cancer treatment [79,80,81,82].

The most exciting recent development in cancer treatment is immunotherapy, which offers tremendous hope and enthusiasm, and attracts numerous ongoing efforts. There has been some compelling successful treatment of blood cancer; however, immunotherapy is not generally useful in solid tumors, and remains somewhat of a disappointment, presenting difficult challenges, particularly in treating gynecologic malignancies [83,84,85,86,87,88].

With so many potentially excellent specific targets, the apparent success of the taxane drugs, which stabilize microtubules, and platinum agents, which by form DNA adducts, over the many other possible mechanisms, is somewhat surprising. The issue of a lack of more expected successes in cancer treatment is most elegantly stated in “The Truth in Small Doses: Why We’re Losing the War on Cancer—and How to Win It”, a book by Clifton Leaf in 2013 [89].

## 4. Resistance of Neoplastic Cells to Programmed Cell Death

We propose that the desensitization of programmed cell death pathways and processes acquired during the malignant progression of cancer cells makes the targeted drugs ineffective.

For the majority of targeted therapies, such as those inhibiting signaling pathways, cellular metabolism, and cell cycle progression, one makes the assumption that disrupting and/or interfering with these cancer-specific targets and pathways will somehow make neoplastic cells less fit and trigger apoptosis programmed cell death [90,91,92,93] (Figure 1). However, we would like to counter that the cellular intrinsic apoptosis programmed cell death pathways are impeded following selection in the process of neoplastic transformation and cancer progression [90,93,94,95,96]. That malignant cells are resistant to programmed cell death seems reasonable and is supported by ample evidence [93,95,96,97,98]. It is likely that neoplastic cells have a higher threshold for activating apoptosis or other programmed cell death pathways [99,100]. Non-transformed normal cells are sensitive to programmed cell death signaling as part of the tissue homeostasis and physiological regulation. Targeting a mutation or alteration in neoplastic cells may reduce their fitness and growth, but will not reach the threshold to trigger the activation of apoptosis, which is key to a strong response and efficacy. Thus, a reduced programmed cell death sensitivity in cancer cells would cause drug resistance and impair the efficacy of the targeted therapies, limiting the power and hope of targeted therapy (Figure 1).

In contrast, based on recent advances in the understanding of the mechanisms, both taxanes and platinum agents kill cancer cells by physical rupturing nuclear membranes, independent of the intrinsic cellular programmed cell death mechanism [101,102].

## 5. Nuclear Membrane Rupture Induced by Taxanes

Taxanes are generally recognized as mitotic inhibitors that stabilize cellular microtubules, leading to mitotic arrest and ultimately mitotic catastrophe [36,103,104,105,106,107], and somehow trigger apoptosis [99,108]. However, the mechanism of cell death induced by taxanes has not been well established and is still mysterious [48]. Several studies have cast doubt on mitotic blockage as a key mechanism of taxanes [109,110,111,112], and non-mitotic mechanisms were suggested or proposed [113,114,115,116,117].

**Figure 2:**
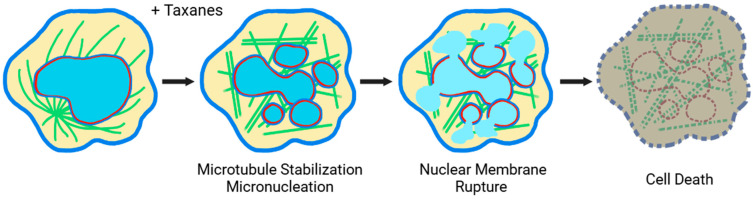
Cell death mechanisms: Taxanes induce micronucleation and irreversible nuclear membrane rupture. An illustration of the cellular mechanism of the microtubule-stabilizing anti-cancer drugs/agents taxanes: the drugs induce microtubule stabilization, micronucleation, and irreversible membrane rupture, leading to a slow and degenerative non-apoptotic cell death process. (blue lines, membrane; green lines, microtubules; red lines, nuclear lamina; light blue area, DNA).

A consistent result was that treatment of cancer cells with paclitaxel and other taxanes results in the generation of micronuclei, termed multinucleation or micronucleation [115,116,117,118,119,120]. Nearly all cancer cells become micronucleated following 48 h of exposure to paclitaxel, prior to eventual elimination [116]. Laboratory studies of cultured cancer cells suggest that paclitaxel stimulates the generation of multiple micronuclei through both mitotic [36,120,121], and non-mitotic [116,117] mechanisms. Further laboratory research results suggest that the malleable nuclear envelope, often due to a loss or reduction of nuclear lamina, Lamin A/C, is more susceptible to breaking and micronucleation in the presence of paclitaxel [116,122]. This property of neoplastic cells–a malleable/fragile nuclear envelope, often due to reduced nuclear lamina [49,123,124,125]–is suggested to be a second specificity/selectivity of taxanes, in addition to their selectivity for the higher proliferation rate in cancer cells [49,122,126]. Thus, in neoplastic cells treated with taxanes, rigid and stabilized microtubule bundles pull apart the malleable/fragile nuclear envelope into multiple micronuclei by physical force [102,117]. Micronuclei are known to be prone to catastrophic and irreversible rupture, leading to cell death [127,128]. The drastic increase in surface area upon micronucleation will likely also stretch the nuclear membrane to a breaking point [102,117]. Thus, the proposed mechanism of taxane-induced cancer cell death suggests that taxanes kill cancer cells by inducing micronucleation and subsequent rupture of micronuclei, rather than triggering a programmed cell death pathway [102,117] (Figure 2). Following the irreversible rupture of the nuclear membrane, the cell death process appears to be a slow decline in the cellular function and structural integrity [102,129].

In sum, recent advances in understanding suggest that taxanes kill cancer cells through the physical rupture of nuclear membranes, rather than the long-thought apoptosis pathway, independent of the intrinsic cellular programmed cell death mechanism [102,117,130].

## 6. Nuclear Membrane Compromising Caused by Platinum Agents

**Figure 3:**
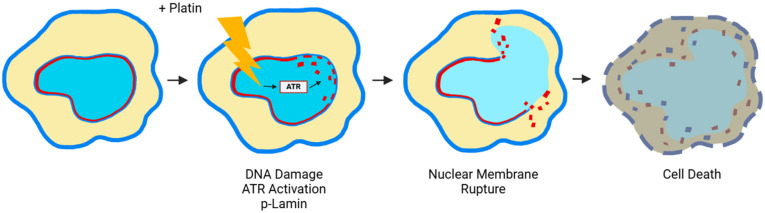
DNA-damaging drugs causes nuclear lamina rupture through activation of ATR to phosphorylate Lamin A/C, leading to its disassembly. An illustration of the proposed mechanism of anti-cancer drug/agent cisplatin/carboplatin with sequential steps: DNA damage, ATR activation, phosphorylation of Lamin A/C, lamina disassembly, and ultimate nuclear membrane rupture and death. (blue lines, membrane; red lines, nuclear lamina; light blue area, DNA).

The platinum compounds (cisplatin/carboplatin/oxaliplatin) also are highly useful and common in treating various types of cancer [1,3,29,32,33,34]. The general concept is that platinum agents kill cancer cells by forming DNA adducts and triggering cell death program [1,3,29,31,32,33,34,131]. However, the mechanism(s) and pathways underlying the action of platinum agents are far from certain [32,33,34]. A recent study proposed that DNA damage caused by platinum agents triggers the activation of ATR, which phosphorylates Lamin A/C, thus causing its disassembly. Subsequently, the nuclear membrane ruptures due to the weakened lamina [101,132,133] (Figure 3). Thus, platinum drugs may kill cancer cells by rupturing the nuclear envelop as well.

## 7. Prospective: Cancer Therapy with Non-Programmed Cell Death Mechanisms and Effects to Reduce or Prevent Side Effects

Therefore, in contrast to the general notion, recent advances in understanding lead to a proposal that both taxanes and platinum agents kill cancer cells by physical rupturing nuclear membranes rather than the long-thought apoptosis, independent of the intrinsic cellular programmed cell death mechanism (Figure 2 and Figure 3).

The new recognition of the non-programmed cell death mechanism in the success of chemotherapeutic agents may inspire oncologists and cancer researchers to direct their efforts towards developing effective future cancer therapies to replace the current standard taxane/platinum regimen [21,22,134].

Cancer chemotherapy is limited by both the unavoidable development of drug resistance and response rate, prompting active efforts to develop strategies to overcome these challenges [32,42,135,136]. Another way to improve chemotherapy is to develop approaches to reduce and prevent side effects [137,138]. The widespread use of taxanes in the course of cancer treatment is often limited by dose-limiting toxicity and intolerable side effects, mainly neutropenia and peripheral neuropathy, rather than a lack of efficacy [137,138,139]. A better understanding of drug mechanisms will likely provide guidance for ways to increase efficacy and avoid resistance. Regarding the second aspect of preventing side effects, the recent discovery [140,141] of the ability of low-intensity ultrasound to neutralize taxane cytotoxicity offers exciting possibilities. Furthermore, new understanding of cancer-killing mechanisms of taxanes [102,117,122] and platinum agents [101,132,133] also provide the opportunity to develop strategies to overcome drug resistance. Additionally, new microtubule-stabilizing agents are under development for drugs with improved drug delivery and increased potency [5,134,142]. With a deeper understanding of cellular actions, platinum and taxane chemotherapy are likely to see progressive improvements over.

At present, no common and practical therapies are on the horizon to replace the classic chemotherapy using platinum agents and taxanes and other microtubule-stabilizing drugs. Therefore, the chemotherapy procedure is likely to be used in oncology for many years to come. However, the lessons learned from the unexpected successes of paclitaxel/platinum chemotherapy and the challenges of targeting therapies will surely enable the great minds among oncologists to design superior cancer treatments, likely based on non-apoptotic mechanisms, bringing us closer to a future where the fear of cancer is no longer a reality.

## 8. Conclusions

The persistent power of taxanes and platinum agents in cancer treatment is ac-counted for by the newly found cellular actions of these agents by physical rupture of nuclear membranes rather than triggering apoptosis, independent of the intrinsic cellular programmed cell death mechanism. Thus, cancer treatment with a non-programmed cell death mechanism of action may be a superior therapy than strategy to induce cancer cell apoptosis, which is desensitized in carcinogenesis.

## Data Availability

Not applicable.

## References

[1] Muggia F. (2009). Platinum compounds 30 years after the introduction of cisplatin: Implications for the treatment of ovarian cancer. Gynecol. Oncol..

[2] Jain A., Dubashi B., Reddy K.S., Jain P. (2011). Weekly paclitaxel in ovarian cancer-the latest success story. Curr. Oncol..

[3] Muggia F.M., Bonetti A., Hoeschele J.D., Rozencweig M., Howell S.B. (2015). Platinum Antitumor Complexes: 50 Years Since Barnett Rosenberg’s Discovery. J. Clin. Oncol..

[4] Bookman M.A. (2016). Optimal primary therapy of ovarian cancer. Ann. Oncol..

[5] Gallego-Jara J., Lozano-Terol G., Sola-Martínez R.A., Cánovas-Díaz M., de Diego Puente T. (2020). A compressive review about taxol: History and future challenges. Molecules.

[6] Kardinal C.G. (1985). Cancer chemotherapy. Historical aspects and future considerations. Postgrad. Med..

[7] Wright J.C. (1984). Cancer chemotherapy: Past, present, and future—Part I. J. Natl. Med. Assoc..

[8] Wright J.C. (1984). Cancer chemotherapy: Past, present, and future—Part II. J. Natl. Med. Assoc..

[9] Papac R.J. (2001). Origins of cancer therapy. Yale J. Biol. Med..

[10] Chabner B.A., Roberts Jr T.G. (2005). Timeline: Chemotherapy and the war on cancer. Nat. Rev. Cancer.

[11] Burchenal J.H. (1977). The historical development of cancer chemotherapy. Semin. Oncol..

[12] Capizzi R.L., Keiser L.W., Sartorelli A.C. (1977). Combination chemotherapy—Theory and practice. Semin. Oncol..

[13] Zubrod C.G., A Schepartz S., Carter S.K. (1977). Historical background of the National Cancer Institute’s drug development thrust. J. Natl. Cancer Inst. Monogr..

[14] DeVita V.T., Chu E. (2008). A history of cancer chemotherapy. Cancer Res..

[15] DeVita Jr V.T., Rosenberg S.A. (2012). Two hundred years of cancer research. N. Engl. J. Med..

[16] Zubrod C.G. (1979). Historic milestones in curative chemotherapy. Semin. Oncol..

[17] Zubrod C.G. (1980). The fifth Myron Karon Memorial Lecture. The cure of cancer by chemotherapy—Reflections on how it happened. Med. Pediatr. Oncol..

[18] Ribatti D. (2012). Sidney Farber and the treatment of childhood acute lymphoblastic leukemia with a chemotherapeutic agent. Pediatr. Hematol. Oncol. J..

[19] Morrison W.B. (2010). Cancer chemotherapy: An annotated history. J. Vet. Intern. Med..

[20] Hajdu S.I. (2005). 2000 years of chemotherapy of tumors. Cancer.

[21] Rowinsky E.K., Donehower R.C. (1991). Taxol: Twenty years later, the story unfolds. J. Natl. Cancer Inst..

[22] Rowinsky E.K., Donehower R.C. (1995). Paclitaxel (taxol). N. Engl. J. Med..

[23] Rosenberg B., Van Camp L., Trosko J.E., Mansour V.H. (1969). Platinum compounds: A new class of potent antitumour agents. Nature.

[24] Hoeschele J.D. (2009). In remembrance of Barnett Rosenberg. Dalton Trans..

[25] Hoeschele J.D. (2014). Biography of professor barnett rosenberg: A tribute to his life and his achievements. Anticancer. Res..

[26] Hoeschele J.D. (2016). Dr Barnett Rosenberg—A Personal Perspective. Dalton Trans..

[27] Rosenberg B., Van Camp L., Krigas T. (1965). Inhibition of Cell Division in Escherichia Coli by Electrolysis Products from a Platinum Electrode. Nature.

[28] Rosenberg B., Van Camp L. (1970). The successful regression of large solid sarcoma 180 tumors by platinum compounds. Cancer Res..

[29] Ghosh S. (2019). Cisplatin: The first metal based anticancer drug. Bioorg. Chem..

[30] Ozols R.F. (1995). Combination regimens of paclitaxel and the platinum drugs as first-line regimens for ovarian cancer. Semin. Oncol..

[31] Elmorsy E.A., Saber S., Hamad R.S., Abdel-Reheim M.A., El-Kott A.F., AlShehri M.A., Morsy K., Salama S.A., Youssef M.E. (2024). Advances in understanding cisplatin-induced toxicity: Molecular mechanisms and protective strategies. Eur. J. Pharm. Sci..

[32] Yang L., Xie H.-J., Li Y.-Y., Wang X., Liu X.-X., Mai J. (2022). Molecular mechanisms of platinum-based chemotherapy resistance in ovarian cancer (Review). Oncol. Rep..

[33] Zhou J., Kang Y., Chen L., Wang H., Liu J., Zeng S., Yu L. (2020). The Drug-Resistance Mechanisms of Five Platinum-Based Antitumor Agents. Front. Pharmacol..

[34] Zoń A., Bednarek I. (2023). Cisplatin in Ovarian Cancer Treatment-Known Limitations in Therapy Force New Solutions. Int. J. Mol. Sci..

[35] Wani M.C., Horwitz S.B. (2014). Nature as a remarkable chemist: A personal story of the discovery and development of taxol. Anticancer Drugs.

[36] Weaver B.A. (2014). How Taxol/paclitaxel kills cancer cells. Mol. Biol. Cell.

[37] Barbuti A.M., Chen Z.-S. (2015). Paclitaxel through the ages of anticancer therapy: Exploring its role in chemoresistance and radiation therapy. Cancers.

[38] McGuire W.P., Rowinsky E.K., Rosenshein N.B., Grumbine F., Ettinger D.S., Armstrong D.K., Donehower R.C. (1989). Taxol: A unique antineoplastic agent with significant activity in advanced ovarian epithelial neoplasms. Ann. Intern. Med..

[39] McGuire W.P. (1993). Taxol: A new drug with significant activity as a salvage therapy in advanced epithelial ovarian carcinoma. Gynecol. Oncol..

[40] Caldas C., McGuire W.P. (1993). Taxol in epithelial ovarian cancer. J. Natl. Cancer Inst. Monogr..

[41] Bookman M.A., Ozols R.F. (1993). Future directions for paclitaxel (TAXOL) in the treatment of ovarian cancer. Semin. Oncol. Nurs..

[42] Mosca L., Ilari A., Fazi F., Assaraf Y.G., Colotti G. (2021). Taxanes in cancer treatment: Activity, chemoresistance and its overcoming. Drug Resist. Updates.

[43] Ozols R.F. (1992). Chemotherapy for advanced epithelial ovarian cancer. Hematol. Oncol. Clin. N. Am..

[44] Schilder R.J., Ozols R.F. (1992). New therapies for ovarian cancer. Cancer Investig..

[45] Hurwitz H.I., McGuire W.P. (1994). Primary chemotherapy in epithelial ovarian cancer. Obstet. Gynecol. Clin. N. Am..

[46] Trimble E.L., Arbuck S.G., McGuire W.P. (1994). Options for primary chemotherapy of epithelial ovarian cancer: Taxanes. Gynecol. Oncol..

[47] Falzone L., Salomone S., Libra M. (2018). Evolution of Cancer Pharmacological Treatments at the Turn of the Third Millennium. Front. Pharmacol..

[48] Blagosklonny M.V., Fojo T. (1999). Molecular effects of paclitaxel: Myths and reality (a critical review). Int. J. Cancer.

[49] Smith E.R., Li Z., Chen Z.-S., Xu X.-X. (2024). Reassessing specificity/selectivity of taxane-based chemotherapy. Cancer Insight.

[50] Mukherjee S. (2010). The Emperor of All Maladies: A Biography of Cancer.

[51] Guchelaar H., Vermes A., Vermes I., Haanen C. (1997). Apoptosis: Molecular mechanisms and implications for cancer chemotherapy. Pharm. World Sci..

[52] Kabore A.F., Johnston J.B., Gibson S.B. (2004). Changes in the apoptotic and survival signaling in cancer cells and their potential therapeutic implications. Curr. Cancer Drug Targets..

[53] Melet A., Song K., Bucur O., Jagani Z., Grassian A.R., Khosravi-Far R. (2008). Apoptotic pathways in tumor progression and therapy. Adv. Exp. Med. Biol..

[54] Plati J., Bucur O., Khosravi-Far R. (2011). Apoptotic cell signaling in cancer progression and therapy. Integr. Biol..

[55] Pistritto G., Trisciuoglio D., Ceci C., Garufi A., D’Orazi G. (2016). Apoptosis as anticancer mechanism: Function and dysfunction of its modulators and targeted therapeutic strategies. Aging.

[56] Carneiro B.A., El-Deiry W.S. (2020). Targeting apoptosis in cancer therapy. Nat. Rev. Cancer.

[57] Strasser A., Vaux D.L. (2020). Cell Death in the Origin and Treatment of Cancer. Mol. Cell.

[58] Chaudhry G.-E., Akim A.M., Sung Y.Y., Muhammad T.S.T. (2022). Cancer and Apoptosis. Methods Mol. Biol..

[59] Tian X., Srinivasan P.R., Tajiknia V., Uruchurtu A.F.S.S., Seyhan A.A., Carneiro B.A., De La Cruz A., Pinho-Schwermann M., George A., Zhao S. (2024). Targeting apoptotic pathways for cancer therapy. J. Clin. Investig..

[60] Røsland G.V., Engelsen A.S.T. (2015). Novel points of attack for targeted cancer therapy. Basic Clin. Pharmacol. Toxicol..

[61] Hernández Borrero L.J., El-Deiry W.S. (2021). Tumor suppressor p53: Biology, signaling pathways, and therapeutic targeting. Biochim. Biophys. Acta.

[62] Cox A.D. (2001). Farnesyltransferase inhibitors: Potential role in the treatment of cancer. Drugs.

[63] Caponigro F. (2002). Farnesyl transferase inhibitors: A major breakthrough in anticancer therapy? Naples, 12 April 2002. Anticancer Drugs.

[64] Downward J. (2003). Targeting RAS signalling pathways in cancer therapy. Nat. Rev. Cancer.

[65] Pegram M., Slamon D. (2000). Biological rationale for HER2/neu (c-erbB2) as a target for monoclonal antibody therapy. Semin. Oncol..

[66] Shepard H.M., Jin P., Slamon D.J., Pirot Z., Maneval D.C. (2008). Herceptin. The Handbook of Experimental Pharmacology.

[67] Xia X., Gong C., Zhang Y., Xiong H. (2023). The History and Development of HER2 Inhibitors. Pharmaceuticals.

[68] Asghar U., Witkiewicz A.K., Turner N.C., Knudsen E.S. (2015). The history and future of targeting cyclin-dependent kinases in cancer therapy. Nat. Rev. Drug Discov..

[69] Sherr C.J., Beach D., Shapiro G.I. (2016). Targeting CDK4 and CDK6: From discovery to therapy. Cancer Discov..

[70] Fassl A., Geng Y., Sicinski P. (2022). CDK4 and CDK6 kinases: From basic science to cancer therapy. Science.

[71] Druker B.J., Tamura S., Buchdunger E., Ohno S., Segal G.M., Fanning S., Zimmermann J., Lydon N.B. (1996). Effects of a selective inhibitor of the Abl tyrosine kinase on the growth of Bcr-Abl positive cells. Nat. Med..

[72] Zubay G., Druker B.J., Talpaz M., Resta D.J., Peng B., Buchdunger E., Ford J.M., Lydon N.B., Kantarjian H., Capdeville R. (2001). Activity of a specific inhibitor of the BCR-ABL tyrosine kinase in the blast crisis of chronic myeloid leukemia and acute lymphoblastic leukemia with the Philadelphia chromosome. N. Engl. J. Med..

[73] McCormick F.K. (2016). Ras protein as a drug target. J. Mol. Med..

[74] McCormick F. (2022). A brief history of RAS and the RAS Initiative. Adv. Cancer Res..

[75] Aguirre A.J., Stanger B.Z., Maitra A. (2024). Hope on the Horizon: A Revolution in KRAS Inhibition Is Creating a New Treatment Paradigm for Patients with Pancreatic Cancer. Cancer Res..

[76] Wei D., Wang L., Zuo X., Maitra A., Bresalier R.S. (2024). A Small Molecule with Big Impact: MRTX1133 Targets the KRASG12D Mutation in Pancreatic Cancer. Clin. Cancer Res..

[77] Duffy M.J., Crown J. (2021). Drugging “undruggable” genes for cancer treatment: Are we making progress?. Int. J. Cancer.

[78] Tang D., Kroemer G., Kang R. (2021). Oncogenic KRAS blockade therapy: Renewed enthusiasm and persistent challenges. Mol. Cancer.

[79] Perurena N., Situ L., Cichowski K. (2024). Combinatorial strategies to target RAS-driven cancers. Nat. Rev. Cancer.

[80] Dilly J., Hoffman M.T., Abbassi L., Li Z., Paradiso F., Parent B.D., Hennessey C.J., Jordan A.C., Morgado M., Dasgupta S. (2024). Mechanisms of Resistance to Oncogenic KRAS Inhibition in Pancreatic Cancer. Cancer Discov..

[81] Reynolds S. Can Chemo Help KRAS Inhibitors Work Better Against Pancreatic Cancer? NCI News & Events, Cancer Currents Blog. 20 August 2024. https://www.cancer.gov/news-events/cancer-currents-blog/2024/pancreatic-cancer-kras-inhibitors-chemotherapy?cid=eb_govdel.

[82] Singhal A., Styers H.C., Rub J., Li Z., Torborg S.R., Kim J.Y., Grbovic-Huezo O., Feng H., Tarcan Z.C. (2024). Classical Epithelial State Drives Acute Resistance to KRAS Inhibition in Pancreatic Cancer. Cancer Discov..

[83] Dana H., Chalbatani G.M., Jalali S.A., Mirzaei H.R., Grupp S.A., Suarez E.R., Rapôso C., Webster T.J. (2021). CAR-T cells: Early successes in blood cancer and challenges in solid tumors. Acta Pharm. Sin. B.

[84] Chen Q., Lu L., Ma W. (2022). Efficacy, Safety, and Challenges of CAR T-Cells in the Treatment of Solid Tumors. Cancers.

[85] Chen T., Wang M., Chen Y., Liu Y. (2024). Current challenges and therapeutic advances of CAR-T cell therapy for solid tumors. Cancer Cell Int..

[86] Guha P., Heatherton K.R., O’connell K.P., Alexander I.S., Katz S.C. (2022). Assessing the Future of Solid Tumor Immunotherapy. Biomedicines.

[87] Vesely M.D., Zhang T., Chen L. (2022). Resistance Mechanisms to Anti-PD Cancer Immunotherapy. Annu. Rev. Immunol..

[88] Sorkhabi A.D., Khosroshahi L.M., Sarkesh A., Mardi A., Aghebati-Maleki A., Aghebati-Maleki L., Baradaran B. (2023). The current landscape of CAR T-cell therapy for solid tumors: Mechanisms, research progress, challenges, and counterstrategies. Front. Immunol..

[89] Leaf C. (2013). The Truth in Small Doses: Why We’re Losing the War on Cancer—And How to Win It.

[90] Gerl R., Vaux D.L. (2005). Apoptosis in the development and treatment of cancer. Carcinogenesis.

[91] Fulda S., Debatin K.-M. (2006). Modulation of apoptosis signaling for cancer therapy. Arch. Immunol. Ther. Exp..

[92] Ziegler D.S., Kung A.L. (2008). Therapeutic targeting of apoptosis pathways in cancer. Curr. Opin. Oncol..

[93] Gogvadze V., Orrenius S., Zhivotovsky B. (2008). Mitochondria in cancer cells: What is so special about them?. Trends Cell Biol..

[94] Hersey P., Zhang X.D. (2003). Overcoming resistance of cancer cells to apoptosis. J. Cell Physiol..

[95] Igney F.H., Krammer P.H. (2002). Death and anti-death: Tumour resistance to apoptosis. Nat. Rev. Cancer.

[96] Igney F.H., Krammer P.H. (2002). Immune escape of tumors: Apoptosis resistance and tumor counterattack. J. Leukoc. Biol..

[97] Gonzalez V.M., Fuertes M.A., Alonso C., Perez J.M. (2001). Is cisplatin-induced cell death always produced by apoptosis?. Mol. Pharmacol..

[98] Huisman C., Ferreira C.G., Bröker L.E., Rodriguez J.A., Smit E.F., Postmus P.E., Kruyt F.A.E., Giaccone G. (2002). Paclitaxel triggers cell death primarily via caspase-independent routes in the non-small cell lung cancer cell line NCI-H460. Clin. Cancer Res..

[99] Bhalla K.N. (2003). Microtubule-targeted anticancer agents and apoptosis. Oncogene.

[100] Ikuta K., Takemura K., Kihara M., Naito S., Lee E., Shimizu E., Yamauchi A. (2005). Defects in apoptotic signal transduction in cisplatin-resistant non-small cell lung cancer cells. Oncol. Rep..

[101] Smolka M.B., Lammerding J. (2023). ATR takes a crack at the nuclear envelope. Mol. Cell.

[102] Xu A.P., Xu L.B., Smith E.R., Fleishman J.S., Chen Z.-S., Xu X.-X. (2024). Cell death in cancer chemotherapy using taxanes. Front. Pharmacol..

[103] Schiff P.B., Fant J., Horwitz S.B. (1979). Promotion of microtubule assembly in vitro by taxol. Nature.

[104] Schiff P.B., Horwitz S.B. (1980). Taxol stabilizes microtubules in mouse fibroblast cells. Proc. Natl. Acad. Sci. USA.

[105] Horwitz S.B. (1994). Taxol (paclitaxel): Mechanisms of action. Ann. Oncol..

[106] Gascoigne K.E., Taylor S.S. (2009). How do anti-mitotic drugs kill cancer cells. J. Cell Sci..

[107] Jordan M.A., Wilson L. (2004). Microtubules as a target for anticancer drugs. Nat. Rev. Cancer.

[108] Ganansia-Leymarie V., Bischoff P., Bergerat J.-P., Holl V. (2003). Signal transduction pathways of taxanes-induced apoptosis. Curr. Med. Chem. Anti-Cancer Agents.

[109] Komlodi-Pasztor E., Sackett D., Wilkerson J., Fojo T. (2011). Mitosis is not a key target of microtubule agents in patient tumors. Nat. Rev. Clin. Oncol..

[110] Komlodi-Pasztor E., Sackett D.L., Fojo A.T. (2012). Inhibitors targeting mitosis: Tales of how great drugs against a promising target were brought down by a flawed rationale. Clin. Cancer Res..

[111] Mitchison T.J. (2012). The proliferation rate paradox in antimitotic chemotherapy. Mol. Biol. Cell.

[112] Yan V.C., Butterfield H.E., Poral A.H., Yan M.J., Yang K.L., Pham C.-D., Muller F.L. (2020). Why great mitotic inhibitors make poor cancer drugs. Trends Cancer.

[113] Fürst R., Vollmar A.M. (2013). A new perspective on old drugs: Non-mitotic actions of tubulin-binding drugs play a major role in cancer treatment. Pharmzie.

[114] Field J.J., Kanakkanthara A., Miller J.H. (2014). Microtubule-targeting agents are clinically successful due to both mitotic and interphase impairment of microtubule function. Bioorg. Med. Chem..

[115] Mitchison T.J., Pineda J., Shi J., Florian S. (2017). Is inflammatory micronucleation the key to a successful anti-mitotic cancer drug?. Open Biol..

[116] Smith E.R., Leal J., Amaya C., Li B., Xu X.-X. (2021). Nuclear Lamin A/C Expression Is a Key Determinant of Paclitaxel Sensitivity. Mol. Cell Biol..

[117] Smith E.R., Xu X.-X. (2021). Breaking malignant nuclei as a non-mitotic mechanism of taxol/paclitaxel. J. Cancer Biol..

[118] Panvichian R., Orth K., Day M.L., Day K.C., Pilat M.J., Pienta K.J. (1998). Paclitaxel-associated multimininucleation is permitted by the inhibition of caspase activation: A potential early step in drug resistance. Cancer Res..

[119] Merlin J.-L., Bour-Dill C., Marchal S., Bastien L., Gramain M.-P. (2000). Resistance to paclitaxel induces time-delayed multinucleation and DNA fragmentation into large fragments in MCF-7 human breast adenocarcinoma cells. Anti-Cancer Drugs.

[120] Zhu Y., Zhou Y., Shi J. (2014). Post-slippage multinucleation renders cytotoxic variation in anti-mitotic drugs that target the microtubules or mitotic spindle. Cell Cycle.

[121] Zasadil L.M., Andersen K.A., Yeum D., Rocque G.B., Wilke L.G., Tevaarwerk A.J., Raines R.T., Burkard M.E., Weaver B.A. (2014). Cytotoxicity of paclitaxel in breast cancer is due to chromosome missegregation on multipolar spindles. Sci. Transl. Med..

[122] Smith E.R., Wang J.-Q., Yang D.-H., Xu X.-X. (2022). Paclitaxel resistance related to nuclear envelope structural sturdiness. Drug Resist. Updates.

[123] Smith E.R., George S.H., Kobetz E., Xu X. (2018). New biological research and understanding of Papanicolaou’s test. Diagn. Cytopathol..

[124] Capo-chichi C.D., Cai K.Q., Smedberg J., Ganjei-Azar P., Godwin A.K., Xu X.X. (2011). Loss of A-type lamin expression compromises nuclear envelope integrity in breast cancer. Chin. J. Cancer.

[125] Capo-Chichi C.D., Cai K.Q., Simpkins F., Ganjei-Azar P., Godwin A.K., Xu X.-X. (2011). Nuclear envelope structural defects cause chromosomal numerical instability and aneuploidy in ovarian cancer. BMC Med..

[126] Xu X.-X., Smith E.R. (2025). The Second Selectivity of Taxanes to Malignant Cells—Nuclear Envelope Malleability. J. Cancer.

[127] Vargas J.D., Hatch E.M., Anderson D.J., Hetzer M.W. (2012). Transient nuclear envelope rupturing during interphase in human cancer cells. Nucleus.

[128] Hatch E.M., Fischer A.H., Deerinck T.J., Hetzer M.W. (2013). Catastrophic nuclear envelope collapse in cancer cell micronuclei. Cell.

[129] Blagosklonny M.V., Robey R., Sheikh M.S., Fojo T. (2002). Paclitaxel-induced FasL-independent apoptosis and slow (non-apoptotic) cell death. Cancer Biol. Ther..

[130] Schimming R., Mason K.A., Hunter N., Weil M., Kishi K., Milas L. (1999). Lack of correlation between mitotic arrest or apoptosis and antitumor effect of docetaxel. Cancer Chemother. Pharmacol..

[131] Boulikas T., Vougiouka M. (2003). Cisplatin and platinum drugs at the molecular level. (Review). Oncol. Rep..

[132] Kovacs M.T., Vallette M., Wiertsema P., Dingli F., Loew D., Nader G.P.d.F., Piel M., Ceccaldi R. (2023). DNA damage induces nuclear envelope rupture through ATR-mediated phosphorylation of lamin A/C. Mol. Cell.

[133] Joo Y.K., Black E.M., Trier I., Haakma W., Zou L., Kabeche L. (2023). ATR promotes clearance of damaged DNA and damaged cells by rupturing micronuclei. Mol. Cell.

[134] Bernabeu E., Cagel M., Lagomarsino E., Moretton M., Chiappetta D.A. (2017). Paclitaxel: What has been done and the challenges remain ahead. Int. J. Pharm..

[135] Sazonova E.V., Kopeina G.S., Imyanitov E.N., Zhivotovsky B. (2021). Platinum drugs and taxanes: Can we overcome resistance?. Cell Death Discov..

[136] Das T., Anand U., Pandey S.K., Ashby C.R., Assaraf Y.G., Chen Z.-S., Dey A. (2021). Therapeutic strategies to overcome taxane resistance in cancer. Drug Resist. Updates.

[137] Marupudi N.I., E Han J., Li K.W., Renard V.M., Tyler B.M., Brem H. (2007). Paclitaxel: A review of adverse toxicities and novel delivery strategies. Expert Opin. Drug Saf..

[138] Visconti R., Grieco D. (2017). Fighting tubulin-targeting anticancer drug toxicity and resistance. Endocr. Relat. Cancer.

[139] Legha S.S., Tenney D.M., Krakoff I.R. (1986). Phase I study of taxol using a 5-day intermittent schedule. J. Clin. Oncol..

[140] Amaya C., Luo S., Baigorri J., Baucells R., Smith E.R., Xu X.-X. (2021). Exposure to low intensity ultrasound removes paclitaxel cytotoxicity in breast and ovarian cancer cells. BMC Cancer.

[141] Amaya C., Smith E.R., Xu X.-X. (2022). Low Intensity Ultrasound as an Antidote to Taxane/Paclitaxel-induced Cytotoxicity. J. Cancer.

[142] Zhao Y., Mu X., Du G. (2016). Microtubule-stabilizing agents: New drug discovery and cancer therapy. Pharmacol. Ther..

